# Mosaic Bovine Viral Diarrhea Virus Antigens Elicit Cross-Protective Immunity in Calves

**DOI:** 10.3389/fimmu.2020.589537

**Published:** 2020-11-12

**Authors:** Neha Sangewar, Wisam Hassan, Shehnaz Lokhandwala, Jocelyn Bray, Rachel Reith, Mary Markland, Huldah Sang, Jianxiu Yao, Bailey Fritz, Suryakant D. Waghela, Karim W. Abdelsalam, Christopher C. L. Chase, Waithaka Mwangi

**Affiliations:** ^1^ Department of Diagnostic Medicine/Pathobiology, Kansas State University, Manhattan, KS, United States; ^2^ Department of Veterinary Pathobiology, Texas A&M University, College Station, TX, United States; ^3^ Department of Veterinary and Biomedical Sciences, South Dakota State University, Brookings, SD, United States

**Keywords:** cattle, bovine viral diarrhea virus, vaccine, efficacy, mosaic antigen, antigen cocktail, cross-protection, neutralizing antibody

## Abstract

Bovine Viral Diarrhea Virus (BVDV) is an important pathogen that plays a significant role in initiating Bovine Respiratory Disease Complex (BRDC) in cattle. The disease causes multi-billion dollar losses globally due to high calf mortality and increased morbidity leading to heavy use of antibiotics. Current commercial vaccines provide limited cross-protection with several drawbacks such as safety, immunosuppression, potential reversion to virulence, and induction of neonatal pancytopenia. This study evaluates two prototype vaccines containing multiple rationally designed recombinant mosaic BVDV antigens for their potential to confer cross-protection against diverse BVDV strains. Genes encoding three novel mosaic antigens, designated E2^123^, NS2-3^1^, and NS2-3^2^, were designed *in silico* and expressed in mammalian cells for the formulation of a prototype protein-based vaccine. The mosaic antigens contain highly conserved protective epitopes from BVDV-1a, -1b, and -2, and included unique neutralizing epitopes from disparate strains to broaden coverage. We tested immunogenicity and protective efficacy of Expi293^TM^-expressed mosaic antigens (293F-E2^123^, 293F-NS2-3^1^, and 293F-NS2-3^2^), and baculovirus-expressed E2^123^ (Bac-E2^123^) mosaic antigen in calves. The Expi293^TM^-expressed antigen cocktail induced robust BVDV-specific cross-reactive IFN-γ responses, broadly neutralizing antibodies, and following challenge with a BVDV-1b strain, the calves had significantly (*p* < 0.05) reduced viremia and clinical BVD disease compared to the calves vaccinated with a commercial killed vaccine. The Bac-E2^123^ antigen was not as effective as the Expi293^TM^-expressed antigen cocktail, but it protected calves from BVD disease better than the commercial killed vaccine. The findings support feasibility for development of a broadly protective subunit BVDV vaccine for safe and effective management of BRD.

## Introduction

Bovine Viral Diarrhea Virus (BVDV) is a single-stranded RNA virus from the genus *Pestivirus* in the family *Flaviviridae* with a 12.5 kb genome that encodes N^pro^; capsid; the E^rns^, E1, and E2 glycoproteins; NS2-3; NS4A-B; and NS5A-B proteins (1, 2). The BVDV is grouped into antigenically distinct genotypes 1 and 2, and cytopathic (CP) and non-cytopathic (NCP) biotypes based on the effect of virus on infected cell cultures (3). Both genotypes are further divided into various sub-genotypes and in the United States BVDV-1b is the predominant sub-genotype (4). The BVDV is one of the major players in causing Bovine Respiratory Disease Complex (BRDC) in cattle worldwide. The damage caused to the cattle industry by the disease every year is estimated to be worth more than a billion dollar due to high calf mortality, increased treatment costs and production losses (5). In cattle, BVDV infection can be acute or persistent with a range of clinical symptoms such as fever, diarrhea, pneumonia, immunosuppression, congenital malformation, and abortion (5, 6). Persistently infected (PI) cattle are chronic virus shedders and therefore, if not diagnosed and culled, they are the main source of BVDV within a herd (7).

Currently, two types of commercial BVDV vaccines are available in the United States, modified-live virus (MLV) and killed virus (KV) vaccines (8). Although majority of the commercial vaccines contain representative BVDV-1 and -2 strains, cross-protective efficacy of the MLV and KV vaccines against heterologous BVDV strains is still limited (7, 9). The MLV vaccines can confer protection after a single vaccination by inducing neutralizing antibody along with CD4^+^ T cell and CD8^+^ cytotoxic T lymphocyte (CTL) responses (10–13). However, there are safety concerns associated with MLV such as immunosuppression, wild-type BVDV contamination of MLV vaccine and potential reversion to virulence (11, 14). The KV vaccines on the other hand do not offer the same level of protective immunity as MLV vaccines without booster dose and require strong adjuvants which may lead to induction of bovine neonatal pancytopenia (15–18).

BVDV is widespread in the United States and Canada, where diverse strains circulate in cattle (1, 3, 19). Despite BVDV vaccination coverage of nearly 80% of the cattle population, prevalence of PI cattle over the years in North America has remained constant implying that the current vaccines are inefficient in eliminating and controlling BVDV infection (20). Limited strain composition of available vaccines has not kept pace with new genetically and antigenically distinct sub-genotypes arising and circulating in cattle herds (9, 19). Thus, there is a need for a more coherent and contemporary proactive vaccine approach to eradicate BVDV since it is evident that the traditional vaccines have been inadequate in providing broad protection.

BVDV-specific CD4^+^ and CD8^+^ T cells have been detected in the infected and protected animals (16, 21, 22). Apart from neutralizing antibodies, CD4^+^ T cells are critical for enhancing the BVDV-specific antibody response and for clearance of infected cells, whereas CD8^+^ T cells can be directly cytotoxic for BVDV-infected cells (23–26). The BVDV E2 and NS2/3 are immunodominant antigens that drive majority of neutralizing antibody and T cell responses and hence, are frequently selected targets for BVDV subunit vaccine development (11, 27–29). Experimental BVDV subunit vaccines involving recombinant E2 glycoprotein can provide some protection in cattle by limiting pyrexia, weight loss, leucopenia and viremia (21, 29–32). However, the protective immunity generated by the monovalent E2 subunit vaccines are mostly against homologous BVDV strains (21, 29–31). In contrast, a multivalent E2 subunit vaccine can provide some level of cross-protection against BVDV-1 and -2 strains (33). The focus of vaccine development efforts has been chiefly on inducing neutralizing antibody response by E2 glycoprotein (21, 30, 33). Some of the latest reports have highlighted the importance of inclusion of NS2 and NS3 in vaccine for induction of protective BVDV-specific T cell responses (27–29). MHC *DR*-restricted T cell epitopes identified from the highly conserved regions of E2 and NS3 are suitable for inclusion in a subunit vaccine (26, 34). Given the heterogeneity of protective antigens among diverse BVDV isolate, mosaic antigens designed based on consensus protein sequences from circulating strains and addition of unique epitopes from disparate strains is likely to result in a chimeric antigen capable of eliciting broad protection (27, 35, 36).

Contemporary computational techniques were used to design three novel mosaic polypeptides consisting of structural and non-structural antigens that are well-conserved among BVDV genotypes along with an array of well characterized epitopes. These epitopes include defined protective neutralizing epitopes, defined and predicted IFN-γ-inducing CD4^+^ T cell and CTL determinants highly conserved across BVDV-1a, -1b, and -2 strains. In addition, unique strain-specific protective epitopes from disparate BVDV strains were included in order to broaden coverage.

In a previous proof-of-concept study, we found that an adenovirus-vectored prototype vaccine conferred better protection than a commercial multivalent MLV vaccine upon challenge with a BVDV-2a strain (27). In the antigen validation study, three novel mosaic antigens, designated E2^123^, NS2-3^1^, and NS2-3^2^ were designed for immunization of calves. The antigens expressed in replication-deficient adenoviruses elicited significantly higher BVDV-specific antibody and T cell responses compared to a commercial MLV vaccine (27). However, since adenovirus vector is a BSL-2 agent with associated safety concerns, we developed recombinant protein-based prototype vaccines as a safer alternative for eliciting cross-protective immune responses in cattle. Two experimental vaccine formulations, one containing mammalian-expressed E2^123^, NS2-3^1^, and NS2-3^2^ mosaic antigens; and another one containing E2^123^ antigen expressed in insect cells were evaluated for their immunogenicity as well as protective efficacy. The immunized calves were challenged with a BVDV-1b (CA401186a) strain that is prevalent in United States and is considered to be a preferable BVDV-1 strain for vaccine efficacy challenge studies (37). The CA401186a is a non-cytopathic BVDV-1b strain which persistently infects cattle leading to pyrexia and immunosuppression, but it doesn’t cause severe gastrointestinal and respiratory disease (37, 38). *In vitro* virus neutralization against representative BVDV-1 and -2 strains was used to evaluate potential for broad protection.

## Materials and Methods

### Generation of BVDV Mosaic Antigen Expression Constructs

Synthetic genes encoding novel BVDV mosaic antigens were designed and validated as previously described (27). Briefly, previously defined protective B- and T-cell epitopes as well as E2 and NS2-3 polypeptide sequences from BVDV strains whose genome sequences were available **(Supplementary Table)** were aligned to generate consensus polypeptides. Where there was no consensus, the most common amino acid was selected and where there was none, a residue from the BVDV-1b strain, the most prevalent isolate, was selected. The data was utilized to design a mosaic chimeric antigen, designated E2^123^, which comprised of consensus sequences of E2 glycoprotein from BVDV-1a, -1b, and -2. In addition, the chimera included unique strain specific neutralizing B cell and T cell epitopes, and a FLAG tag.

Two mosaic chimeric antigens, NS2-3^1^ and NS2-3^2^, representing diverse NS2-3 antigen repertoire from BVDV-1 and -2, respectively, were similarly designed with a FLAG tag as described above. The novel mosaic polypeptide sequences were used to generate synthetic genes codon-optimized for protein expression in mammalian cells (GenScript). A gene encoding an irrelevant antigen, designated TMSP (Theileria Modified Sporozoite Protein), was also generated and used as a negative control. Expression and authenticity of the proteins encoded by the synthetic genes were validated using BVDV-specific sera, mAbs, and T cells (27).

### Expression and Purification of Recombinant BVDV Mosaic Antigens

The three *flag*-tagged synthetic genes encoding E2^123^, NS2-3^1^, and NS2-3^2^ mosaic antigens were subcloned into pcDNA3.1+ mammalian expression vector (Invitrogen^TM^), which had been modified by addition of a CD5 secretory signal sequence (39). Positive clones for each construct were identified by PCR screening, sequence-verified and subsequently used for recombinant protein expression in the mammalian Expi293^TM^ Expression System (Gibco) as per manufacturer’s protocol and as previously described (40). Briefly, Expi293 cell suspension cultures were transfected with pcDNA3.1+ constructs expressing the mosaic antigens whereby, cell lysate and culture supernatant were combined for purification of E2^123^, whereas NS2-3^1^ and NS2-3^2^ were purified from cell lysate. Anti-FLAG M2 affinity gel (Sigma, A2220) was used for affinity purification of the recombinant mosaic antigens. The gene encoding TMSP was used to similarly generate a FLAG-tagged negative control antigen.

The synthetic gene encoding the E2^123^ mosaic antigen was also subcloned into pFastBac^TM^/HBM-TOPO® vector for baculovirus protein expression (Bac-to-Bac® HBM TOPO® Secreted Expression System, Invitrogen^TM^). Selected positive clones were verified by DNA sequencing and then used to assemble recombinant baculovirus for recombinant protein production using High Five™ insect cell suspension cultures according to manufacturer’s protocol. The antigen was affinity purified from High Five™ cell lysate and culture supernatant using anti-FLAG M2 affinity gel as described above.

### Validation of Purified BVDV Mosaic Antigens

The affinity purified antigens were quality control validated by SDS-PAGE and Western Blotting. The baculovirus-expressed E2^123^, Expi293^TM^-expressed E2^123^, and NS2-3^1^ antigens were resolved in a NuPAGE® Bis-Tris gel (Invitrogen^TM^, NP0322) by denaturing electrophoresis. The gel was then stained with Imperial™ Protein Stain (Invitrogen^TM^, 24615) for visualization of the protein bands. The antigens were resolved on gel as above and transferred to Amersham^TM^ Protran^TM^ 0.45µm Nitrocellulose Membrane (GE Healthcare Life Science, 10600114) by electrophoresis for Western Blot analysis. After transfer, the blot was incubated in blocking buffer, 10% non-fat dry milk in TBST, overnight at 4°C, and then probed for 1 h with anti-BVDV polyclonal sera (Porcine origin, Cat. #210-70-BVD, VMRD, Inc) diluted at 1:3,000 in blocking buffer. Following 3X washes with TBST, the blot was incubated with peroxidase-conjugated goat anti-porcine IgG (Jackson ImmunoResearch, Cat. #114-035-003) diluted at 1:5,000 in blocking buffer. SuperSignal West Pico PLUS substrate (Thermo Scientific, Prod #34577) was used for protein band visualization on immunoblot by chemiluminescence.

### Immunization of Calves

Twenty, four-month old Holstein calves were determined as BVDV sero-negative using the standard serum neutralization assay against BVDV-1 and -2 (Kansas State Veterinary Diagnostic Lab.). The calves were then randomly divided into four groups A–D (n=5) as shown in **Table 1**. Following acclimatization for 10 days, the treatment and control calves were primed at day 0 and then boosted on day 21 with doses as shown in **Table 1**. Each calf in group A was immunized intramuscularly in the neck area with a cocktail of the Expi293^TM^-expressed E2^123^, NS2-3^1^, and NS2-3^2^ formulated in MONTANIDE^TM^ ISA 201 VG adjuvant (Seppic). Similarly, calves in group B received the baculovirus-expressed E2^123^ formulated in the same adjuvant. Calves in group C served as positive controls and were immunized with a commercial BVDV Killed Vaccine (Vira Shield^TM^ 6; Disclaimer: The commercial vaccine was used off label as booster dose was administered at day 21 instead of at day 28–35), whereas calves in group D served as negative controls and were immunized intramuscularly with an irrelevant antigen, TMSP formulated in the MONTANIDE^TM^ ISA 201 VG adjuvant. During immunization, calves were housed together in outdoor pens.

**Table 1:** Calf immunization protocol.

**Groups**	**Calf ID**	**Immunogen**	**Prime-Boost Dose/Calf**
**A: 293F-Cocktail**	39 56 60 65 93	Expi293^TM^-expressed mosaic antigens: E2^123^; NS2-3^1^ and NS2-3^2^	293F-E2^123^: 250 µg 293F-NS2-3^1^: 75 µg 293F-NS2-3^2^: 50 µg
**B: Bac-E2^123^**	99 1 16 9 11	Baculovirus-expressed mosaic antigen: Bac-E2^123^	250 µg
**C: Vira Shield^TM^ 6**	90 92 24 86 6	Commercial BVDV KV vaccine: Vira Shield^TM^ 6	5 ml
**D: Sham**	58 76 21 22 31	Expi293^TM^-expressed irrelevant antigen: TMSP	250 µg

### Evaluation of BVDV-Specific IFN-γ Responses

Antigen-specific IFN-γ responses by peripheral blood mononuclear cells (PBMCs) isolated from blood collected at two weeks post-prime and one week post-boost were evaluated by Enzyme-linked immunospot (ELISPOT) assay using Bovine IFN-γ ELISpot^BASIC^ (ALP) kit (Mabtech; product code: 3119-2A) as per manufacturer’s instruction and as previously described (41). Briefly, 0.125×10^6^ PBMCs were seeded in triplicate wells of MultiScreen-IP plates (MilliporeSigma™ MAIPS4510) and incubated at 37°C for 48 h with 2.5 µg/ml of affinity-purified mosaic antigens, defined BVDV CD4^+^ T cell epitope peptides, representative whole heat-killed BVDV-1b (CA0401186a, TGAC), or BVDV-2a (A125, 1373) strains in a final volume of 100 µl complete RPMI 1640 medium. The positive control was 2.5 µg/ml ConA, whereas medium alone was used as a negative control. The spots were counted with an ELISpot reader [Cellular Technology Limited (CTL) ImmunoSpot® S6 Analyzer] and the results were presented as the mean number of IFN-γ^+^ spot-forming cells (SFC) per 10^6^ PBMCs after background medium counts were deducted.

### Evaluation of Antibody Responses

Antigen-specific IgG responses were determined by indirect ELISA using sera from blood collected before immunization, two weeks post-prime, and three weeks post-boost. Briefly, triplicate wells in polystyrene 96-well microplates were coated overnight at 4°C with 100 µl of affinity purified antigens diluted at 5 µg/ml in bicarbonate coating buffer. Expi293^TM^-expressed mosaic antigens (E2^123^, NS2-3^1^, and NS2-3^2^) were used to evaluate antibody responses in the calves immunized with the Expi293^TM^-expressed antigen cocktail, the Vira Shield^TM^ 6 vaccine, and the negative controls, whereas baculovirus-expressed E2^123^ antigen was used to test sera from calves immunized with the cognate antigen. The plates were washed with PBS containing 0.1% Tween 20 and incubated with 200 µl of 10% sodium caseinate blocking buffer for 1 h at 37°C. 100 µl of sera diluted in blocking buffer (1:500 dilution for pre-bleed and post-prime sera, and 1:5,000 for post-boost sera) were added in triplicates and then incubated at 37°C for 1 h. After washing, 100 μl of 1:5,000 dilution of peroxidase-conjugated goat anti-bovine IgG (Jackson ImmunoResearch, Cat #101-035-003) was added. Plates were incubated for 1 h at 37°C, washed and then developed with Sure Blue Reserve TMB substrate (KPL, Cat# 53-00-02). 1N Hydrochloric acid was used to stop the reactions and the plates were read at 450 nm in BioTek microplate reader (Synergy H1 Multi-mode reader). Antigen-specific IgG responses in sera from the Vira Shield^TM^ 6 vaccinees and the negative controls were also tested at 1:250 dilution for pre-bleed and post-prime sera, and at 1:2,500 for post-boost sera as described above. Antigen-specific IgG responses were presented as mean OD (Optical Density) absorbance for each treatment and control groups.

### Virus Neutralization Assays

Sera from blood collected at three weeks post-boost were tested to determine BVDV-1 and -2 neutralizing antibody titers using BVDV-1 strains (BJ, CA401186a, Singer, NADL, and TGAC) and BVDV-2a strains (A125, 890, 1373, 296 C, and 296 NC) as previously described (27, 42). Briefly, sera was heat-inactivated at 56°C for 30 min, and 50 μl of each serum was serially diluted two-fold in 96-well microtiter plates using minimum essential media (MEM). Fifty micro liters of stock BVDV virus containing 300 TCID_50_/ml was added to each test well. In each test, a positive control serum was also included. The serum/virus mixture was incubated for 1 h at 37°C followed by addition of MDBK cells, and the plates were incubated at 37°C for 72 h. The cells were monitored daily for signs of CPE in cells exposed to cytopathic strains, whereas the presence of non-cytopathic virus strains was detected by immuno-peroxidase assay (43). The results were presented as virus neutralization titers (VNT).

### Animal Challenge

At day 21 post-boost (day 42 post-prime), all the calves were challenged intranasally with BVDV-1b CA0401186a strain. Each calf received 5 ml of 1×10^6^ TCID_50_/ml of the virus in 0.9% saline (37). The inoculum (2.5 ml) was delivered in each nostril using LMA® MAD Nasal™ Intranasal Mucosal Atomization Device (Teleflex; Item number: MAD100). The animals were monitored for reaction to the challenge virus and post-challenge rectal temperatures were recorded daily. Three major clinical outcomes associated with the challenge strain, BVDV-1b CA0401186A, were used for evaluation of the level of protection in calves: pyrexia, leukopenia and viremia in blood; as described previously (37, 38). Challenge study was conducted in ABSL-2 facility where calves were segregated in pens according to their assigned groups (**Table 1**).

### Determination of Viremia and WBC Counts

Post-challenge, blood samples were collected in vacutainer tubes containing EDTA on day 0, 2, 3, 6, 9, 13, and 15 for evaluation of viremia and white blood cell (WBC) counts. Blood samples were lysed by freeze-thawing, centrifuged, and the lysate was used for BVDV isolation to determine viral titer by alkaline phosphatase monolayer immunostaining as previously described (6, 44, 45). Briefly, serial 10-fold dilutions of sample lysate were prepared in Dulbecco’s modified Eagle medium (DMEM) and 50 μl were added to 96-well plate containing fresh MDBK cells. Following incubation at 37°C for 72 h, the cells were fixed for staining with anti-BVDV E2 mAb (Cat. # 348, VMRD) and alkaline phosphatase-conjugated goat anti-mouse IgG (Jackson ImmunoResearch, Cat #115-055-146). BVDV titers in blood samples were reported as the lowest dilution at which the lysate-exposed MDBK cells stained positive for BVDV E2. The profile of CBC in each blood sample was evaluated by HESKA Veterinary Hematology System (RTI LLC, Brookings, SD 57006, USA) with counting parameters set for bovine WBC, HGB, RBC, and PLT. The counts for platelets, WBC, and RBC and WBC morphology were verified microscopically.

### Statistical Analysis

The significance of the differences between the treatments and the controls was determined by ordinary one-way Analysis of Variance (ANOVA) followed by Tukey’s multiple comparison test. Post-immunization, the significance of the differences in BVDV-specific immune responses (IFN-γ responses, IgG responses, and VN titers) were compared among all groups. Post-challenge, mean viral titers for blood viremia were also analyzed by performing comparisons among all groups. However, post-challenge clinical outcomes: mean rectal temperatures and WBCs change ratios were analyzed by performing comparisons between the treatments (293F-Cocktail, Bac-E2^123^, and Vira Shield^TM^ 6), and the negative control group (TMSP sham treatment) by ordinary one-way Analysis of Variance (ANOVA) followed by Dunnett’s multiple comparison test. Statistical analysis was performed using GraphPad Prism 7 (Version 7.04, GraphPad Software, Inc. La Jolla, CA) and a significance level of *p* < 0.05 was used for all analyses.

## Results

### Design and Expression of Novel Recombinant BVDV Mosaic Antigens

**Figure 1:**
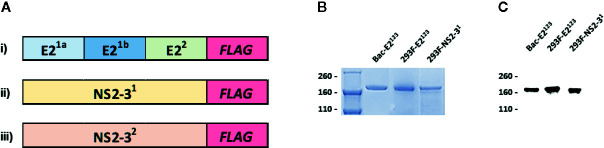
Recombinant BVDV Mosaic Antigens. **(A)** Schematic diagram of codon-optimized synthetic genes encoding novel BVDV mosaic antigens: i) E2^123^ contains mosaic genes: E2^1a^, E2^1b^ and E2^2^ encoding consensus sequences of E2 glycoprotein from BVDV-1a, -1b and -2, respectively; ii) NS2-3^1^; and iii) NS2-3^2^ encodes for mosaic BVDV-1 and -2 nonstructural proteins 2-3, respectively. A gene encoding the FLAG tag was added in-frame at the end of the synthetic genes for affinity purification of the recombinant antigens; **(B)** SDS-PAGE; and **(C)** Western Blot analyses of the affinity-purified baculovirus-expressed E2^123^ (Bac-E2^123^), Expi293^TM^-expressed E2^123^ (293F-E2^123^) and NS2-3^1^ (293F-NS2-3^1^) antigens probed with anti-BVDV polyclonal serum generated against BVDV-1 and -2 strains. The molecular weights are expressed in kDa. The Expi293^TM^-expressed NS2-3^2^ (293F-NS2-3^2^) is not shown.

Three pCDNA3 constructs encoding novel BVDV mosaic antigens (E2^123^; NS2-3^1^; and NS2-3^2^) were used to express recombinant antigens by transient transfection of Expi293F cells (**Figure 1A**). Baculovirus encoding the E2^123^ mosaic polypeptide was also used to generate recombinant protein using High Five™ cells. Affinity-purified recombinant antigens were validated by SDS-PAGE (**Figure 1B**) and Western Blot using BVDV-1- and -2-specific polyclonal serum (**Figure 1C**).

### Mosaic BVDV Antigens Induced Strong IFN-γ Responses

**Figure 2:**
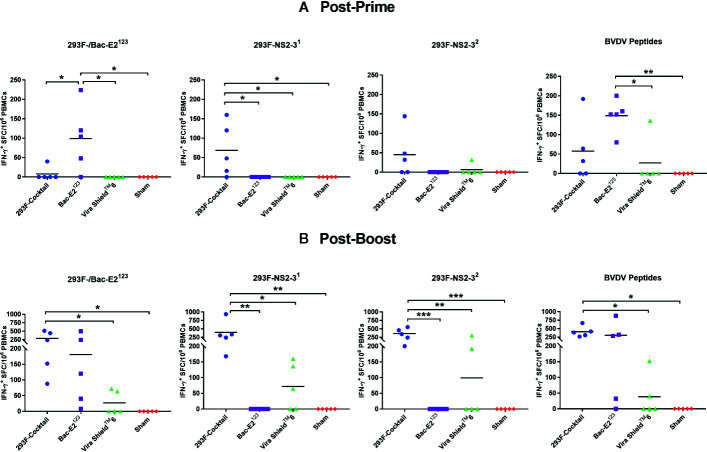
BVDV mosaic antigen-specific IFN-γ responses. IFN-γ secreting PBMC responses against recombinant BVDV mosaic antigens and defined BVDV CD4^+^ T cell epitopes were determined at two weeks post-prime **(A)** and one week post-boost **(B)** by IFN-γ ELISPOT assays. The response is presented as IFN-γ^+^ SFC/10^6^ PBMCs. For E2^123^ antigen-specific IFN-γ readouts, PBMCs from 293F-Cocktail, Vira Shield^TM^ 6 and Sham groups were stimulated with 293F-E2^123^ antigen whereas PBMCs from Bac-E2^123^ group were stimulated with the Bac-E2^123^ antigen. Medium alone served as the negative control and the data shown is minus media background counts. The group mean is represented by a bar. Asterisks denote statistically significant differences between the groups (*p < 0.05, **p < 0.01 and ***p < 0.001).

Immunogenicity and protective efficacy of immunogens formulated using the recombinant mosaic antigens was evaluated in calves following prime-boost immunization (**Table 1**). After priming, antigen-specific IFN-γ responses were detected in calves immunized with the 293F-expressed antigen cocktail (E2^123^, NS2-3^1^, and NS2-3^2^) and in the calves immunized with the baculovirus-expressed E2^123^antigen (**Figure 2A**). Notably, the Bac-E2^123^-immunized calves had a significantly higher (*p* < 0.05) post-prime E2^123^-specific IFN-γ response compared to the calves immunized with the 293F-expressed antigen cocktail and the calves immunized with the Vira Shield^TM^ 6 commercial vaccine (**Figure 2A**). Strong IFN-γ response against NS2-3^1^ was only detected in the calves immunized with the 293F-expressed antigen cocktail and the response was significantly higher (*p* < 0.05) than the response detected in the calves immunized with the Vira Shield^TM^ 6 vaccine (**Figure 2A**). The calves immunized with the 293F-expressed antigen cocktail also had a high mean NS2-3^2^-specific IFN-γ response, but this response was not significantly different from the other treatment groups (**Figure 2A**). Following priming, no E2^123^- and NS2-3^1^-specific IFN-γ responses were detected in the calves immunized with the Vira Shield^TM^ 6 vaccine and only 1/5 calf in this group had IFN-γ response against NS2-3^2^ (**Figure 2A**).

Boosting expanded antigen-specific IFN-γ responses in the calves immunized with the 293F-expressed antigen cocktail and the calves immunized with the Bac-E2^123^ antigen (**Figure 2B**). The calves in both treatment groups had high levels of E2^123^-specific IFN-γ responses, but only the mean response of the calves immunized with the 293F-expressed antigen cocktail was significantly higher (*p* < 0.05) than the response detected in the Vira Shield^TM^ 6 and the sham control groups (**Figure 2B**). The calves immunized with the 293F-expressed antigen cocktail also had the strongest NS2-3^1^- and NS2-3^2^-specific IFN-γ responses (**Figure 2B**). Significantly higher NS2-3^1^-specific IFN-γ response was detected in the calves immunized with the 293F-expressed antigen cocktail compared to the responses detected in the calves immunized with the Vira Shield^TM^ 6 vaccine (*p* < 0.05) and the sham treatment (*p* < 0.01) (**Figure 2B**). Boosting with the 293F-expressed antigen cocktail significantly expanded NS2-3^2^-specific response compared to the Vira Shield^TM^ 6 vaccine (*p* < 0.01) and the sham treatment (*p* < 0.001) (**Figure 2B**). Overall, post-boost antigen-specific responses were significantly low in the calves immunized with the Vira Shield^TM^ 6 vaccine where only 2/5, 3/5, and 2/5 calves responded to E2^123^, NS2-3^1^, and NS2-3^2^, respectively (**Figure 2B**).

IFN-γ responses were also analyzed using a peptide pool containing previously defined BVDV CD4^+^ T cell epitopes (34) (**Figure 2**). Calves immunized with the 293F-expressed antigen cocktail and the Bac-E2^123^ exhibited high levels of post-prime CD4^+^ T cell epitope-specific IFN-γ responses (**Figure 2A**). The Bac-E2^123^ antigen elicited significantly higher epitope-specific responses compared to Vira Shield^TM^ 6 vaccine (*p* < 0.05) and sham treatment (*p* < 0.01) (**Figure 2A**). The epitope-specific IFN-γ responses were evidently amplified post-boost in the calves immunized with either the 293F-expressed antigen cocktail or the Bac-E2^123^ antigen (**Figure 2B**). However, the calves immunized with the 293F-expressed antigen cocktail had significantly higher (*p* < 0.05) response than the other treatment and control groups (**Figure 2B**). Interestingly, post-boost IFN-γ responses against the defined BVDV CD4^+^ T cell epitopes induced by Vira-Shield™ 6 were considerably lower, with only 2/5 calves responding, than the responses induced by the prototype vaccines (**Figure 2B**).

### Mosaic Antigens Induced Robust Cross-Reactive BVDV-Specific IFN-γ Responses

**Figure 3:**
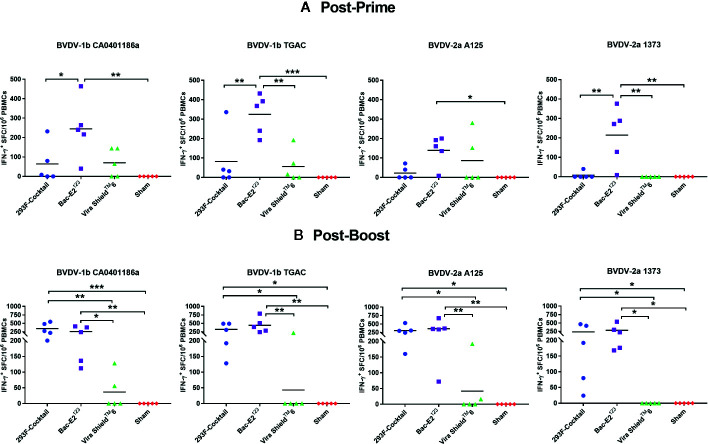
BVDV-1 and -2 specific IFN-γ responses. IFN-γ secreting PBMC responses against BVDV-1b and -2a strains were determined at two weeks post-prime **(A)** and one week post-boost **(B)** by IFN-γ ELISPOT assays. The response is presented as IFN-γ^+^ SFC/10^6^ PBMCs. Medium alone served as the negative control and the data shown is minus media background counts. The group mean is represented by a bar. Asterisks denote statistically significant differences between the groups (*p < 0.05, **p < 0.01 and ***p < 0.001).

The 293F-expressed antigen cocktail and the Bac-E2^123^ antigen, but not the Vira Shield^TM^ 6 vaccine, primed and expanded IFN-γ responses that were recalled in the majority of the vaccinees by representative BVDV-1b and -2a strains (**Figure 3**). Following priming, the Bac-E2^123^ antigen, but not the 293F-expressed antigen cocktail, elicited IFN-γ memory responses that were consistently recalled by representative BVDV-1b strains (CA0401186a and TGAC) and BVDV-2a strains (A125 and 1373) (**Figure 3A**). However, following boosting, the memory responses expanded by the 293F-expressed antigen cocktail and the Bac-E2^123^ antigen, but not the Vira Shield^TM^ 6 vaccine, were consistently recalled by the representative BVDV-1b and -2a strains and in addition, the magnitude of the recall responses were similar (**Figure 3B**). These post-boost responses recalled by the representative BVDV-1b and -2a strains were significantly (*p* < 0.05) higher than the recall responses detected in calves immunized with the Vira Shield^TM^ 6 vaccine (**Figure 3B**). The IFN-γ memory responses induced by the Vira Shield^TM^ 6 vaccine were low and poorly recalled by the representative BVDV-1b and -2a strains whereby, post-boost recall responses were detected only in 2/5 for CA0401186a, 1/5 for TGAC, 2/5 for A125, and none for 1373 (**Figure 3B**).

### Mosaic Antigens Induced BVDV Cross-Neutralizing Antibodies

**Figure 4:**
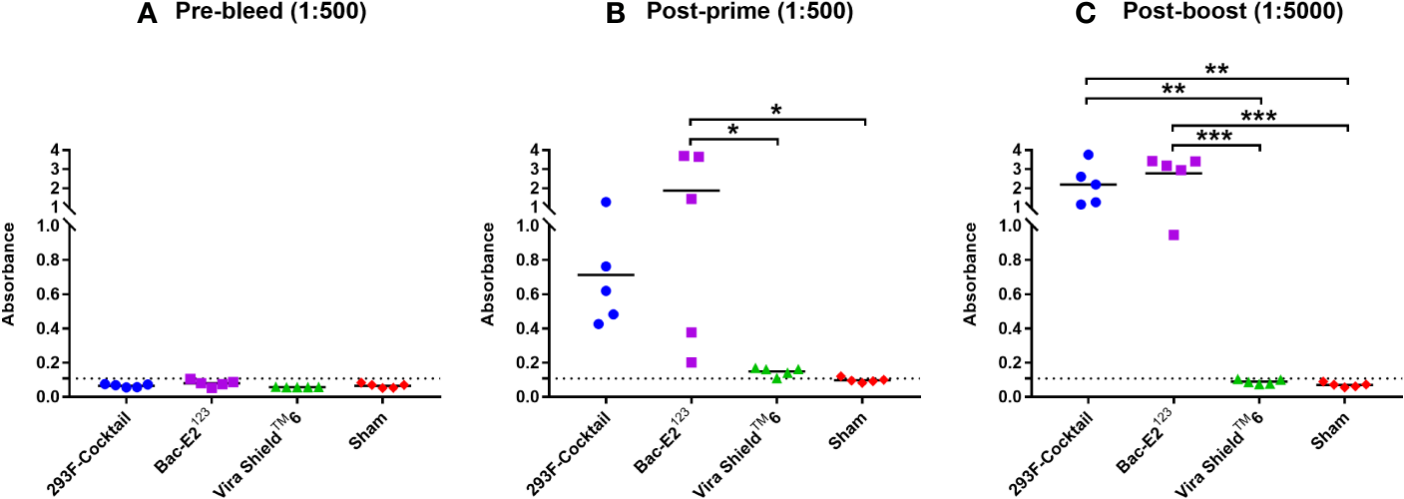
BVDV mosaic antigen-specific IgG responses. IgG responses against recombinant BVDV mosaic antigens were determined using serum samples collected prior to immunization **(A)**, at two weeks post-prime **(B)** and at three weeks post-boost **(C)** by ELISA. The group mean is represented by a bar. Asterisks denote statistically significant differences between the groups (*p < 0.05, **p < 0.01 and ***p < 0.001). IgG responses in Vira Shield^TM^ 6 group were also determined at lower dilutions: 1:250 and 1:2500 for post-prime and post-boost respectively where the average absorbance detected were 0.509 and 0.410 respectively (not shown in figure).

Immunization of calves with the recombinant antigens induced strong antibody responses that were significantly amplified after boosting (**Figure 4**). After priming, all the calves immunized with the prototype vaccines seroconverted (**Figures 4A, B**). Both the 293F-expressed antigen cocktail and Bac-E2^123^ antigen primed high antigen-specific IgG responses, but the mean IgG response induced by Bac-E2^123^ antigen was significantly higher (*p* < 0.05) than the response induced by the Vira Shield^TM^ 6 vaccine and the sham treatment (**Figure 4B**). Post-boost IgG responses recalled in the calves immunized with the 293F-expressed antigen cocktail (*p* < 0.01) and the Bac-E2^123^ antigen (*p* < 0.001) were significantly higher than the responses recalled in calves immunized with the Vira Shield^TM^ 6 vaccine and the sham treatment calves (**Figure 4C**).

**Figure 5:**
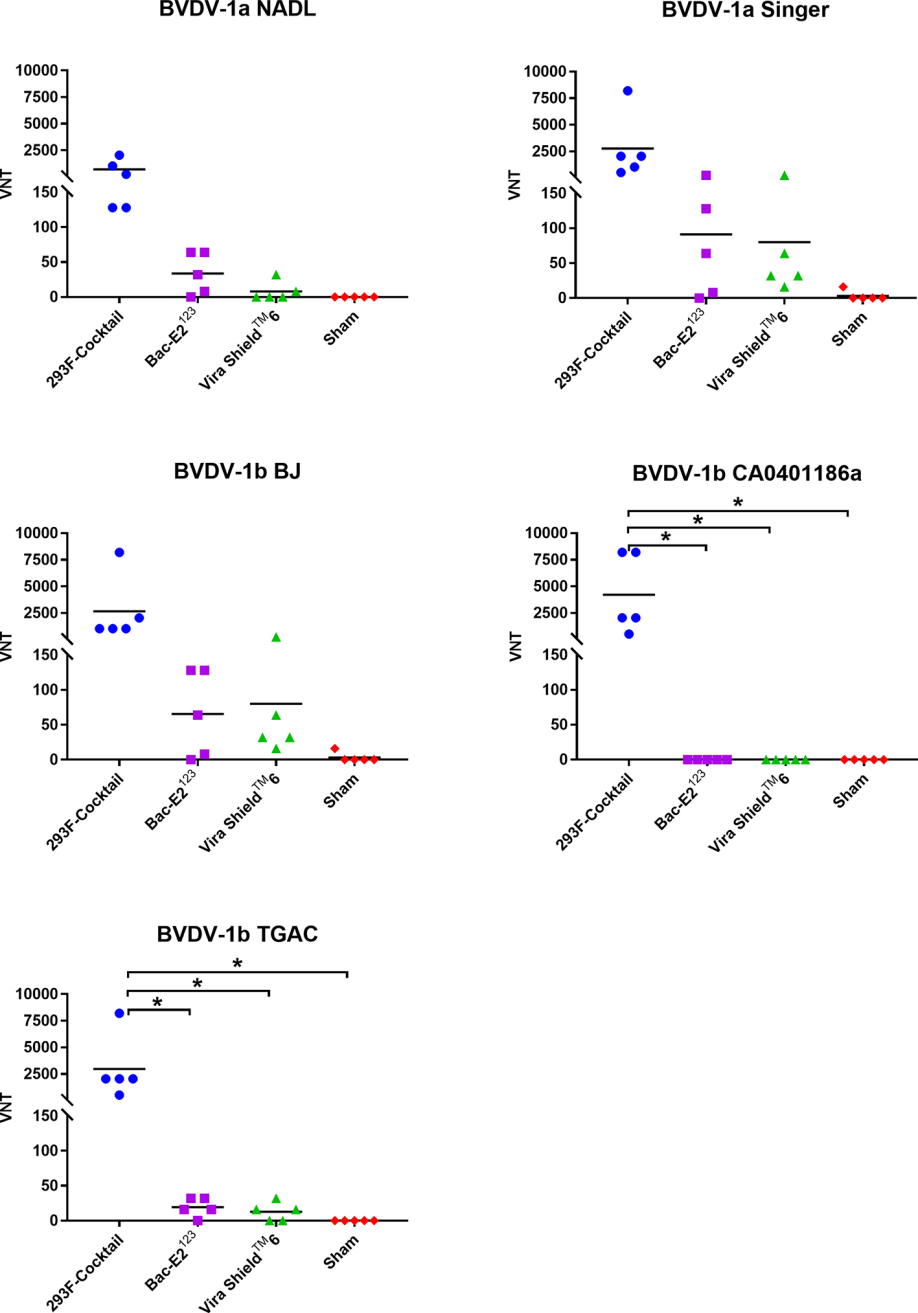
BVDV-1 specific neutralizing antibody titers. Virus neutralization assays were used to evaluate BVDV-1-specific neutralization titers in immunized calves at three weeks post-boost against the representative BVDV-1a and -1b strains. Mean group virus neutralization titers (VNT) are represented by the bars. Asterisks denote statistically significant differences between the groups (*p < 0.05).

Virus neutralizing (VN) antibodies against BVDV-1 strains were detected in the immunized calves three weeks post-boost (**Figure 5**). The 293F-expressed antigen cocktail elicited the highest levels of VN titers (1:128 to 1:8192) against the five BVDV-1 strains that were detected in all the vaccinees (**Figure 5**). However, compared to the other treatment groups, mean VN titer for the 293F-expressed antigen cocktail group was significantly higher (*p* < 0.05) for two BVDV-1b strains, CA0401186a and TGAC (**Figure 5**). The Bac-E2^123^ antigen elicited BVDV-1-specific VN titers (1:8 to 1:256) in most of the vaccinees against BVDV-1a NADL, BVDV-1a Singer, BVDV-1b BJ and BVDV-1b TGAC, but there was no detectable BVDV-1b CA0401186a-specific neutralizing antibody response (**Figure 5**). On the other hand, all calves immunized with the Vira Shield^TM^ 6 vaccine had detectable but low VN titers (1:16 to 1:256) against BVDV-1a Singer and BVDV-1b BJ, the BVDV-1 strains included in the Vira Shield™ 6 vaccine (**Figure 5**). Additionally, VN titers (1:8 to 1:32) induced by the Vira Shield^TM^ 6 vaccine against BVDV-1a NADL (2/5 calves) and BVDV-1b TGAC (3/5 calves) were lower compared to the responses induced by the 293F-expressed antigen cocktail and the Bac-E2^123^ antigen (**Figure 5**). The Vira Shield^TM^ 6 vaccine did not induce detectable neutralizing antibody response against BVDV-1b CA0401186a, which was similar to the outcome observed in the calves immunized with the Bac-E2^123^ antigen (**Figure 5**). Altogether, the 293F-expressed antigen cocktail induced broader and consistent VN antibody responses against BVDV-1 strains (**Figure 5**).

**Figure 6:**
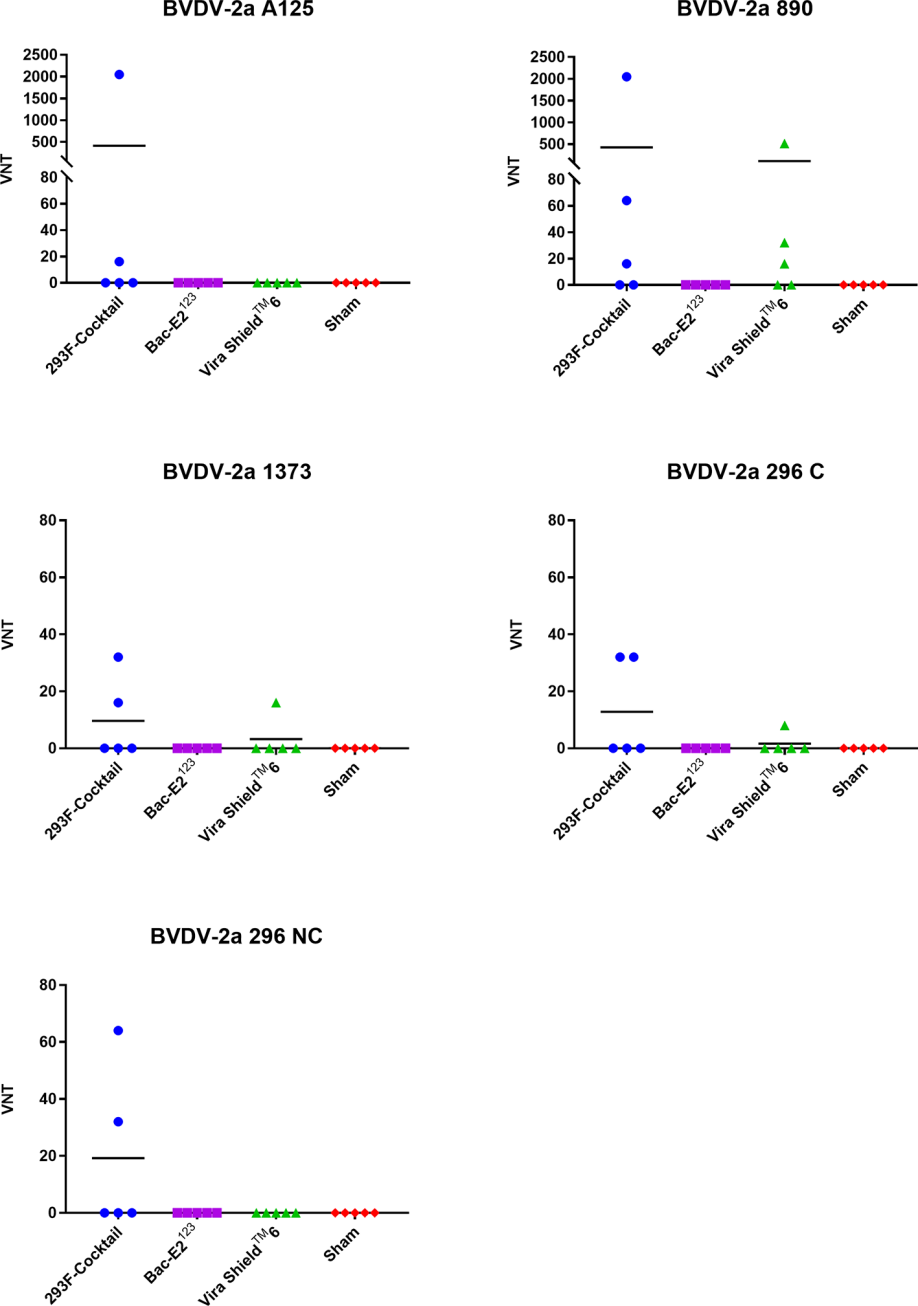
BVDV-2-specific neutralizing antibody titers. Virus neutralization assays were used to evaluate BVDV-2-specific neutralization titers in immunized calves at three weeks post-boost against the representative BVDV-2a strains. Mean group virus neutralization titers (VNT) are represented by the bars.

The 293F-expressed antigen cocktail elicited VN antibodies against all five BVDV-2a strains in either 2/5 or 3/5 vaccinees, but the responses were inconsistent and generally low (except two responses against A125 and 890 strains) compared to the responses against BVDV-1 strains (**Figure 6**). Surprisingly, the Bac-E2^123^ antigen did not induce detectable VN antibodies against any of the BVDV-2a strains (**Figure 6**). The Vira Shield^TM^ 6 vaccine induced VN antibodies against 890 (3/5 calves), 1373 (1/5 calves), and 296 C (1/5 calves) strains, however the responses were poor except the response by one calf (1:512) mounted against the 890 strain (**Figure 6**). Similar to the BVDV-1 specific VN responses, BVDV-2 VN antibody responses induced by the 293F-expressed antigen cocktail were better than the responses induced by the Bac-E2^123^ antigen and the Vira Shield^TM^ 6 vaccine (**Figure 6**).

### Mosaic Antigens Conferred Protection Against BVDV-1b

**Figure 7:**
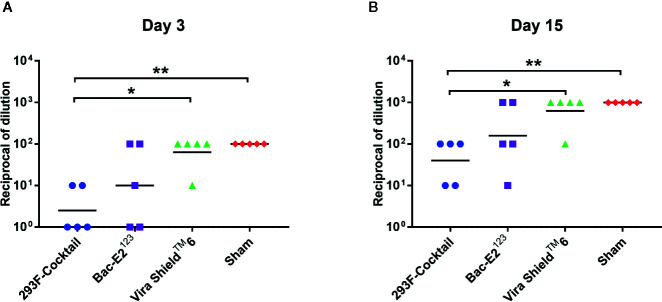
Post-challenge viremia in calves challenged with BVDV-1b (CA0401186a). Viremia detected in blood samples collected from calves on day 3 **(A)** and day 15 **(B)** post-challenge. Mean group dilutions are represented by the bars. Asterisks denote statistically significant differences between the groups (*p < 0.05 and **p < 0.01).

Three days post-challenge, the calves immunized with the 293F-expressed antigen cocktail or the Bac-E2^123^ antigen had lower mean BVD virus titers compared to the Vira Shield^TM^ 6 vaccinees and the negative controls (**Figure 7A**). Specifically, the 293F-expressed antigen cocktail vaccinees had significantly lower mean viremia compared to the Vira Shield^TM^ 6 vaccinees (*p* < 0.05) as well as the negative controls (*p* < 0.01) (**Figure 7A**). The mean viremia for the 293F-expressed antigen cocktail vaccinees was also lower than that observed in the calves immunized with the Bac-E2^123^ antigen, but the difference was not significant (**Figure 7A**). Notably, 3/5 of the calves immunized with the 293F-expressed antigen cocktail and 2/5 of calves immunized with the Bac-E2^123^ antigen had no viremia three days post-challenge (**Figure 7A**). But thereafter, all the calves had viremia on days 6–13 (data not shown) and even though the mean viremia for all the treatment groups and the negative controls was higher on day 15 post-challenge, the trend was consistent with the outcome observed on day 3 post-challenge (**Figure 7B**). There was no difference in mean viremia between the Vira Shield^TM^ 6 vaccinees and the negative controls at 3 and 15 days post-challenge (**Figure 7**).

**Figure 8:**
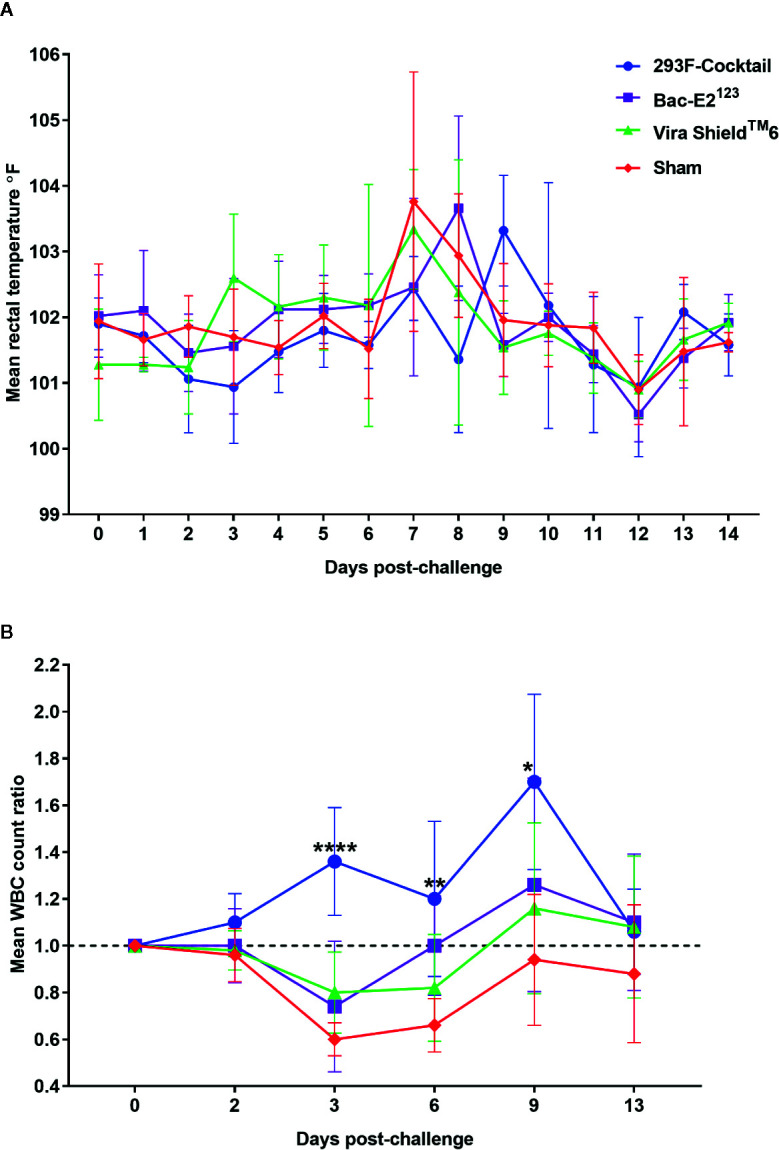
Post-challenge clinical outcomes. **(A)** Mean rectal temperature fluctuation. **(B)** Mean change ratios of white blood cell counts in treatment and control groups. Asterisks denote statistically significant differences as compared to the negative controls (*p < 0.05, **p < 0.01 and ****p < 0.0001).

Following challenge, all the calves had fever but there were overt differences in temperature fluctuation patterns between the treatment groups (**Figure 8A**). Notably, the calves immunized with the 293F-expressed antigen cocktail or the Bac-E2^123^ antigen had delayed temperature peak whereby the highest mean temperature peaked on days 9 and 8, respectively (**Figure 8A**). However, no significant difference in post-challenge mean temperatures were detected among the treatment and control groups (**Figure 8A**). The calves immunized with the Vira Shield^TM^ 6 vaccine had biphasic pyrexia typical of BVDV infection with first peak in mean body temperature on day 3 followed by a higher peak on day 7 (**Figure 8A**). The negative control calves also had fever and their mean body temperature peaked on day 7 (**Figure 8A**).

The most dramatic outcome, post-challenge, was the observation that the calves immunized with the 293F-expressed antigen cocktail never experienced leukopenia and had increased mean WBCs counts that were significantly higher than the negative control group on day 3 (*p* < 0.0001), day 6 (*p* < 0.01), and day 9 (*p* < 0.05) (**Figure 8B**). In contrast, the calves in all the other treatment groups as well as the negative controls had leukopenia 3 days post-challenge (**Figure 8B**). The calves immunized with the Bac-E2^123^ antigen recovered by day 6, whereas the Vira Shield^TM^ 6 vaccinees recovered by around day 8, but the negative controls had not recovered by day 13 (**Figure 8B**).

## Discussion

There is still a need for safe and more efficacious vaccines for protection of cattle against diverse BVDV strains. Three novel mosaic BVDV polypeptides designated E2^123^, NS2-3^1^, and NS2-3^2^, which consist of immunogenic antigens highly conserved among BVDV-1 and -2 strains were generated and evaluated for their potential to induce cross-protection against diverse BVDV strains. This approach has previously been pursued to generate cross-protective vaccine candidates for pathogens with heterogeneous circulating strains or subtypes (27, 35, 36). In a previous proof-of-concept study, the mosaic polypeptides induced BVDV-specific antibody and T cell responses and conferred protection against a BVDV-2 strain following immunization with adenovirus expression constructs (27). In the current study, the genes encoding the three mosaic polypeptides were used to express recombinant proteins in Human Embryonic Kidney Expi293F cells (E2^123^, NS2-3^1^, and NS2-3^2^) or High Five™ insect cells (E2^123^) and authenticity of the affinity purified antigens was confirmed using polyclonal serum generated against BVDV-1 and -2 strains (**Figure 1**) (27). Immunogenicity and protective efficacy of two prototype vaccines formulated using a cocktail of the 293F-expressed antigens or the Bac-E2^123^ antigen was evaluated by prime-boost immunization of calves followed by challenge with a BVDV-1b strain, the predominant sub-genotype in United States (4).

Both prototype vaccines, but not the Vira Shield^TM^ 6 commercial vaccine, primed strong IFN-γ responses against the immunizing antigens and the induced memory was recalled by peptides generated using well characterized *DRB*-restricted BVDV CD4^+^ T cell epitope sequences (**Figure 2A**) (26, 34). This suggests that priming with a single dose of the prototype vaccines could generate memory responses that can be recalled upon BVDV infection. This outcome was further supported by the observation that, the IFN-γ memory induced by the 293F-expressed antigen cocktail underwent strong recall after boosting (**Figure 2B**). Moreover, these outcomes were consistent with the previous findings where the same mosaic antigens expressed by recombinant adenoviruses elicited IFN-γ responses of similar magnitude that were strongly recalled by BVDV CD4^+^ T cell epitopes in the immunized calves but not in the commercial MLV vaccinees (27).

Experimental BVDV subunit vaccines induce strong IFN-γ responses against the immunizing antigens (21, 29, 31) however, there is very limited evidence as to whether these responses are recalled against BVDV (27, 33). The antigen-specific IFN-γ responses elicited by the prototype vaccines were strongly and consistently recalled by all the representative BVDV-1b (CA0401186a and TGAC) and BVDV-2a (A125 and 1373) strains (**Figure 3**). The BVDV-1b and -2a strain-specific IFN-γ recall responses detected in the calves immunized with the prototype vaccines were also significantly (*p*<0.05) higher than the recall responses detected in the Vira Shield^TM^ 6 vaccinees (**Figure 3B**). The pro-inflammatory anti-viral response of IFN-γ is crucial for limiting BVDV infection in cattle (46, 47). Therefore, the potential of 293F-expressed antigen cocktail and the Bac-E2^123^ antigen to elicit strong BVDV-specific IFN-γ memory responses is of great significance as it could result in improved vaccine efficacy.

Cattle infected with BVDV develop neutralizing antibodies against the virus (48, 49). The prototype vaccines generated high levels of BVDV antigen-specific IgG responses in the immunized calves (**Figure 4**). The elicited IgG responses, especially by the 293F-expressed antigen cocktail, contributed towards BVDV-1a, -1b, and -2a neutralization as demonstrated by the detected VN titers against diverse strains (**Figures 5**, **6**). Importantly, the calves immunized with the 293F-expressed antigen cocktail developed exceptionally high VN titers (in the range of 1:1024 to >1:8192) against the representative BVDV-1 strains (**Figure 5**). Neutralizing antibody titers of this magnitude are usually achieved with MLV vaccination or multiple BVDV exposures (9, 43). Additionally, these titers were higher than the BVDV-1 neutralizing antibody titers that were generated in calves immunized with recombinant adenoviruses expressing the mosaic antigens (27). In contrast, the Bac-E2^123^ antigen and Vira Shield^TM^ 6 vaccine induced moderate to very low BVDV-1 neutralizing antibody titers in calves (**Figure 5**). The 293F-expressed antigen cocktail induced BVDV-2a neutralizing antibodies in a few calves and the overall titers were inferior compared to the BVDV-1 neutralizing antibody titers (**Figure 6**). This outcome was similar to a previous finding in which, following boosting with recombinant adenoviruses expressing the mosaic antigens, low BVDV-2a neutralizing antibody titers were detected in 3/5 immunized calves but all 5/5 calves were completely protected upon challenge with a BVDV-2a strain (27). The Vira Shield^TM^ 6 vaccine elicited either no or very low BVDV-2a neutralizing antibody titers, whereas the Bac-E2^123^ antigen failed to elicit BVDV-2a neutralizing antibodies in calves (**Figure 6**). It is worth noting that altogether, the prototype vaccines induced higher neutralizing antibody titers against BVDV-1 strains than BVDV-2 strains. Recombinant adenoviruses expressing the mosaic antigens had also induced better BVDV-1 neutralizing antibody titers than BVDV-2 neutralizing antibody titers (27). Since the three components of the E2^123^ mosaic antigen were generated using E2 from BVDV-1a, -1b, and -2, epitope coverage was biased towards BVDV-1 genotype, and thus it can be deduced that the skewed neutralizing antibody response towards BVDV-1 strains is likely due to the presence of higher number of BVDV-1 neutralizing epitopes than the BVDV-2 neutralizing epitopes in the mosaic antigens. The data from *in vitro* virus neutralization demonstrate that the prototype vaccine containing the 293F-expressed antigen cocktail is better than the Vira Shield^TM^ 6 vaccine in eliciting broadly neutralizing antibodies and therefore, has the potential to bridge the gap between the protective immunity conferred by the MLV and KV vaccines (9, 15, 38).

Potency of the immune responses elicited in calves by the prototype vaccines was reflected by clinical outcomes following challenge with BVDV-1b. Compared to the Vira Shield^TM^ 6 vaccine, the immune responses induced by the 293F-expressed antigen cocktail had significant (*p*<0.05) effect on the onset of viremia as indicated by the absence (3/5) and very low titers (2/5) of virus in the vaccinees on day 3 post-challenge (**Figure 7A**). Although not as effective as the immune responses induced by the 293F-expressed antigen cocktail, the immune responses elicited by the Bac-E2^123^ antigen also inhibited the onset of BVDV infection in 2/5 calves (**Figure 7A**). All the calves had BVDV later during the challenge, but the 293F-expressed antigen cocktail significantly (*p*<0.05) reduced viremia in calves compared to the Vira Shield^TM^ 6 vaccine (**Figure 7B**). The Vira Shield^TM^ 6 vaccine, concurrent with previous reports (18, 50), was unsuccessful in limiting viremia and therefore, the vaccinated calves had similar level of viremia as the negative control calves (**Figure 7**).

The two prototype vaccines also protected calves from BVD disease better than the Vira Shield^TM^ 6 vaccine by significantly reducing the disease outcomes. There was no fever detected in the calves immunized with the 293F-expressed antigen cocktail until day 9 post-challenge, compared to fever peaking on day 8 in calves immunized with the Bac-E2^123^ antigen, whereas fever in calves vaccinated with the Vira Shield^TM^ 6 vaccine peaked on day 7 concurrently with the negative control calves (**Figure 8A**). Most importantly, following challenge, the calves immunized with the 293F-expressed antigen cocktail were protected from immunosuppression (**Figure 8B**). In comparison, the calves immunized with the Bac-E2^123^ antigen had mild leukopenia, whereas the calves immunized with the Vira Shield^TM^ 6 vaccine had significant decrease in WBC counts from day 3 until around day 8 post-challenge (**Figure 8B**). This outcome indicates that the 293F-expressd antigen cocktail can elicit better immune responses for improved BRD management in cattle.

In this study, rationally designed prototype BVDV vaccines performed significantly better compared to a traditional commercial vaccine by effectively priming broad BVDV-specific IFN-γ and neutralizing antibody responses that were strongly recalled upon boost. However, the 293F-expressed antigen cocktail and the Bac-E2^123^ antigen conferred strikingly different levels of protection in cattle. Non-structural antigen NS3 when used as an immunogen by itself confers protection in cattle by significantly reducing viremia (28, 51) since it induces T cell responses important for controlling the BVDV infection (25, 29, 34). Moreover, NS3 in Flavivirus is a highly conserved non-structural antigen critical for inducing protective T cell responses and this could explain why the 293F-expressed antigen cocktail, which contained the NS2-3 antigen, was more efficacious at reducing the viral burden as well as preventing immunosuppression in vaccinees compared to the Bac-E2^123^ antigen (52–54).

The cross-neutralizing antibody responses elicited by the two prototype vaccines were also significantly different. The 293F-expressed antigen cocktail induced antibody responses that were better at *in vitro* BVDV cross-neutralization. Compared to the 293F-expressed antigen cocktail, the Bac-E2^123^ antigen, despite inducing high E2-specific IgG responses, elicited lower BVDV-1 neutralizing antibody titers with no BVDV-2 neutralization. The n-glycosylation pattern in Pestivirus E2 glycoprotein is relevant to the protein structure and function, and hence, to the infectivity of virus (55–57). Similar to our findings, BVDV E2 antigen produced in insect cells have been previously demonstrated to elicit BVDV neutralizing antibodies however, it doesn’t confer complete protection in cattle (30, 33, 58, 59). Therefore, it could be concluded that the inherently simpler post-translational modifications offered by the insect cells influence the antigenicity of candidate vaccine in a manner that potentially leads to misrepresentation of some, if not all, key conformational neutralizing epitopes (30, 60, 61). Mammalian expression system, on the other hand, generates more authentic post-translationally modified antigen and thereby is more suitable for the production of an efficacious subunit vaccine (21, 30, 32, 61).

In conclusion, the results presented here demonstrate that the mosaic BVDV antigens conferred broader and better protection than a current commercial vaccine. Therefore, a targeted approach of designing a computationally optimized vaccine for broader coverage can be developed and deployed to improve management of BVDV in cattle. Furthermore, this study highlights and reinforces the impact of the non-structural antigens on vaccine efficacy. The Flavivirus non-structural antigens, which are relatively more conserved compared to E2, are known to be rich in broadly protective T cell epitopes that have been exploited in order to improve vaccine efficacy (54, 62–64). Therefore, in future the BVDV non-structural antigens apart from NS2-3, need to be investigated to identify other protective determinants for inclusion in a contemporary subunit vaccine. The outcomes from this pilot study also provide insight into the gaps in current vaccines’ efficacy that warrants future BVDV vaccine upgrades.

## Data Availability Statement

The original contributions presented in the study are included in the article/**Supplementary Material**. Further inquiries can be directed to the corresponding authors.

## Ethics Statement

The animal study was reviewed and approved by Kansas State University Institutional Animal Care and Use Committee and Institutional Biosafety Committee.

## Author Contributions

WM and SW designed and oversaw the development and characterization of the experimental vaccines. The vaccines were generated in lab by NS, WH, RR, MM, SL, and JB. Animal immunization, sample collection and challenge experiments were done by NS, SL, WM, RR, MM, HS, and JY. KA and CC conducted serum neutralization assays. NS and BF performed IFN-γ ELISPOT and viremia assays. NS and WM were involved in data analysis, result interpretation and drafted the initial manuscript. All authors contributed to the article and approved the submitted version.

## Funding

This work was supported by the Agriculture and Food Research Initiative Competitive Grant number 2015-06984 from the USDA National Institute of Food and Agriculture.

## Conflict of Interest

The authors declare that the research was conducted in the absence of any commercial or financial relationships that could be construed as a potential conflict of interest.

## Abbreviations

293F, Expi293^TM^-expressed; Bac, Baculovirus-expressed; E2^123^, Mosaic antigen containing E2 glycoprotein from BVDV-1a, -1b and -2; NS2-3^1^, Mosaic nonstructural 2-3 antigen from BVDV-1; NS2-3^2^, Mosaic nonstructural 2-3 antigen from BVDV-2; TMSP, Theileria Modified Sporozoite Protein.
