# Supplementary material for: Mosaic Bovine Viral Diarrhea Virus Antigens Elicit Cross-Protective Immunity in Calves
Source: Front Immunol. 2020 Nov 12;11:589537. doi: 10.3389/fimmu.2020.589537 (PMC7690067; doi:10.3389/fimmu.2020.589537)
Supplement: Supplementary file 1 [file Table_1.docx]

**Supplementary Table.** BVDV genomes used for designing the mosaic BVDV antigens.

| Genotype | BVDV Strain | Genbank Accession Number |
| --- | --- | --- |
| BVDV-1a | Singer | [DQ088995.2](https://www.ncbi.nlm.nih.gov/nuccore/DQ088995.2) |
|  | NADL | [M31182.1](https://www.ncbi.nlm.nih.gov/nuccore/M31182.1) |
|  | 6010 | [JN380080.1](https://www.ncbi.nlm.nih.gov/nuccore/JN380080.1) |
|  | 8844 | [HQ174293.1](https://www.ncbi.nlm.nih.gov/nuccore/HQ174293.1) |
|  | 180 | [HQ174292.1](https://www.ncbi.nlm.nih.gov/nuccore/HQ174292.1) |
|  | 08GB44-1 | [JQ418633.1](https://www.ncbi.nlm.nih.gov/nuccore/JQ418633.1) |
|  | SD-1 | [M96751.1](https://www.ncbi.nlm.nih.gov/nuccore/M96751.1) |
|  | Oregon C24V | [AF091605.1](https://www.ncbi.nlm.nih.gov/nuccore/AF091605.1) |
|  | Nose | [AB033752.1](https://www.ncbi.nlm.nih.gov/nuccore/AB033752.1) |
| BVDV-1b | Hercules | [JX297517.1](https://www.ncbi.nlm.nih.gov/nuccore/JX297517.1) |
|  | RK-13 | [JX419398.1](https://www.ncbi.nlm.nih.gov/nuccore/JX419398.1) |
|  | Powder | [JN380089.1](https://www.ncbi.nlm.nih.gov/nuccore/JN380089.1) |
|  | PJ | [JN380088.1](https://www.ncbi.nlm.nih.gov/nuccore/JN380088.1) |
|  | CC13B | [KF772785.1](https://www.ncbi.nlm.nih.gov/nuccore/KF772785.1) |
|  | 8824 | [HQ174295.1](https://www.ncbi.nlm.nih.gov/nuccore/HQ174295.1) |
|  | 6151 | [JN380083.1](https://www.ncbi.nlm.nih.gov/nuccore/JN380083.1) |
|  | 12F004 | [KC963967.1](https://www.ncbi.nlm.nih.gov/nuccore/KC963967.1) |
|  | 8830 | [HQ174296.1](https://www.ncbi.nlm.nih.gov/nuccore/HQ174296.1) |
|  | CP7 | [U63479.1](https://www.ncbi.nlm.nih.gov/nuccore/U63479.1) |
|  | Columba | [JX297514.1](https://www.ncbi.nlm.nih.gov/nuccore/JX297514.1) |
|  | Mars | [JX297520.1](https://www.ncbi.nlm.nih.gov/nuccore/JX297520.1) |
|  | Osloss | [M96687.1](https://www.ncbi.nlm.nih.gov/nuccore/M96687.1) |
|  | NY-1 | [AY027671.1](https://www.ncbi.nlm.nih.gov/nuccore/AY027671.1) |
|  | Hastings | [AF083349.1](https://www.ncbi.nlm.nih.gov/nuccore/AF083349.1) |
| BVDV-2 | PI99 | [JN380086.1](https://www.ncbi.nlm.nih.gov/nuccore/JN380086.1) |
|  | AM1 | [JN380085.1](https://www.ncbi.nlm.nih.gov/nuccore/JN380085.1) |
|  | NRW 19-13-8_Dup(-) | [HG426489.1](https://www.ncbi.nlm.nih.gov/nuccore/HG426489.1) |
|  | NRW 19-13-1_Dup(-) | [HG426487.1](https://www.ncbi.nlm.nih.gov/nuccore/HG426487.1) |
|  | D37-13-2_Dup(-) | [HG426479.1](https://www.ncbi.nlm.nih.gov/nuccore/HG426479.1) |
|  | NRW 12-13_Dup(-) | [HG426483.1](https://www.ncbi.nlm.nih.gov/nuccore/HG426483.1) |
|  | NRW 14-13_Dup(-) | [HG426485.1](https://www.ncbi.nlm.nih.gov/nuccore/HG426485.1) |
|  | D75-13-609_Dup(-) | [HG426481.1](https://www.ncbi.nlm.nih.gov/nuccore/HG426481.1) |
|  | VOE 4407 | [HG426495.1](https://www.ncbi.nlm.nih.gov/nuccore/HG426495.1) |
|  | Parker | [AF145971.1](https://www.ncbi.nlm.nih.gov/nuccore/AF145971.1) |
|  | 296nc | [AF145969.1](https://www.ncbi.nlm.nih.gov/nuccore/AF145969.1) |
|  | 125c | [AF083345.1](https://www.ncbi.nlm.nih.gov/nuccore/AF083345.1) |
|  | Potsdam 1600 | [HG426491.1](https://www.ncbi.nlm.nih.gov/nuccore/HG426491.1) |
|  | C413 | [AF002227.1](https://www.ncbi.nlm.nih.gov/nuccore/AF002227.1) |
|  | 1373 | [AF145967.2](https://www.ncbi.nlm.nih.gov/nuccore/AF145967.2) |
|  | New York'93 | [AF502399.1](https://www.ncbi.nlm.nih.gov/nuccore/AF502399.1) |
|  | SH-28 | [HQ258810.1](https://www.ncbi.nlm.nih.gov/nuccore/HQ258810.1) |
|  | NRW 19-13-8_Dup(+) | [HG426490.1](https://www.ncbi.nlm.nih.gov/nuccore/HG426490.1) |
|  | NRW 12-13_Dup(+) | [HG426484.1](https://www.ncbi.nlm.nih.gov/nuccore/HG426484.1) |
|  | 37621 | [HQ174303.1](https://www.ncbi.nlm.nih.gov/nuccore/HQ174303.1) |
|  | 793 | [HQ174302.1](https://www.ncbi.nlm.nih.gov/nuccore/HQ174302.1) |
|  | IAF-103 | [HQ174301.1](https://www.ncbi.nlm.nih.gov/nuccore/HQ174301.1) |
|  | 890 | [U18059.1](https://www.ncbi.nlm.nih.gov/nuccore/U18059.1) |
|  | SH2210-14 | [HG426492.1](https://www.ncbi.nlm.nih.gov/nuccore/HG426492.1) |
|  | p24515 | [AY149216.1](https://www.ncbi.nlm.nih.gov/nuccore/AY149216.1) |
